# GLIM Criteria for Assessment of Malnutrition in Saudi Patients with Type 2 Diabetes

**DOI:** 10.3390/nu15040897

**Published:** 2023-02-10

**Authors:** Sondos Albukhari, Mahmoud M. A. Abulmeaty, Abdullah M. Alguwaihes, Mustafa Shoqeair, Dara Aldisi, Adel Alhamdan

**Affiliations:** 1 Community Health Sciences Department, College of Applied Medical Sciences, King Saud University, Riyadh 11362, Saudi Arabia; 2 Department of Medicine, College of Medicine, King Saud University, Riyadh 12372, Saudi Arabia; 3 Clinical Nutrition Department, King Khalid University Hospital, King Saud University Medical City, Riyadh 12372, Saudi Arabia

**Keywords:** GLIM, malnutrition, type 2 diabetes, validation

## Abstract

The Global Leadership Initiative on Malnutrition (GLIM) is a new approach established for the assessment of malnutrition. This study aimed to validate the GLIM for the diagnosis of malnutrition in patients with type 2 diabetes mellitus (T2DM) in Saudi Arabia, using the Subjective Global Assessment (SGA) as a reference. In addition, the association between the GLIM criteria and vascular complications in those patients was examined. A cross-sectional study was conducted on 101 patients with T2DM. The level of agreement between the GLIM and SGA tools was calculated using the kappa coefficient (κ). A receiver operating characteristic curve was used to determine the sensitivity and specificity of the GLIM. In addition, binary logistic regression was performed to investigate the association between each GLIM criterion and T2DM vascular complications. According to both the GLIM and the SGA, malnutrition was found in 15.8% and 17.8% of patients, respectively. The GLIM criteria achieved a very good level of accuracy (AUC = 0.877). The agreement between the tools was substantial (κ = 0.778). The ‘disease/inflammation’ criterion of the GLIM was significantly associated with macrovascular complications. To conclude, the GLIM criteria for diagnosis of malnutrition presented satisfactory levels of validity, and as such are acceptable for assessing the nutritional status of patients with T2DM.

## 1. Introduction

Diabetes mellitus is a common metabolic disease characterized by prolonged hyperglycemia, which impacts both physical and psychosocial health. There is a high prevalence of diabetes across all regions of the world, with an estimated 537 million adults worldwide having the disease [1]. The International Diabetes Federation ranks the Kingdom of Saudi Arabia (KSA) among the top 10 nations for diabetes prevalence, with a projected rate of 20.8% by 2030 [2]. Type 2 diabetes mellitus (T2DM) accounts for around 90% of all diabetes cases worldwide and is associated with both macrovascular and microvascular complications [3]. 

Malnutrition has been defined by the World Health Organization [4] as a deficiency, excess, or imbalance between essential nutrient supplies and energy. It is a complication that can occur in adult patients with diabetes, regardless of their body mass index (BMI). Malnutrition is affected by various factors and, because of its complications and comorbidities in diabetes, the nutritional status of patients with diabetes can deteriorate [5].

Screening and assessing the nutritional status of patients with T2DM is uncommon. This may be due to the absence of a universally accepted consensus on the definition of malnutrition in patients with T2DM. For most of the patients with T2DM who visit outpatient or inpatient departments, the focus of their healthcare providers may be on managing blood glucose levels and acute diseases, rather than malnutrition.

Over the past decades, several nutritional screening tools have been introduced. The Nutrition Risk Screening 2002 (NRS-2002) is a tool recommended by the European Society for Clinical Nutrition and Metabolism (ESPEN) for detecting nutritional risks in hospital settings [6]. It is considered a valid tool for assessing malnutrition risk [7,8,9]. The Subjective Global Assessment (SGA), developed by Detsky et al., is also used as a clinical tool to assess nutritional status [10]. It is highly regarded, universally, and is widely used to identify disease-related malnutrition because it is safe, simple, and inexpensive. Although it has some limitations, it is considered the gold standard method against which other nutritional assessment tools should be validated [11].

Recently, the Global Leadership Initiative on Malnutrition (GLIM) convened [12], gathering the major global clinical nutrition societies—the American Society for Parenteral and Enteral Nutrition (ASPEN), the European Society for Clinical Nutrition and Metabolism (ESPEN), the Federacion Latinoamericana de Terapia Nutricional, the Nutricion Clínica y Metabolismo (FELANPE), and the Parenteral and Enteral Nutrition Society of Asia [PENSA]—to standardize malnutrition criteria in adults, and in doing so create a globally agreed-upon tool for the diagnosis of malnutrition in different clinical settings. The recommended GLIM approach comprises two steps. The first step is risk screening to identify patients at risk of malnutrition using any validated screening tool, including the NRS-2002, and the second step is the diagnosis of malnutrition. The GLIM criteria are a consensus that needs to be validated before it can be approved for use in clinical practice. There is little research regarding the validity of the GLIM criteria in T2DM. To address this, we hypothesized that GLIM is a comparable tool for diagnosing malnutrition among Saudi patients with T2DM in comparison to the SGA and that GLIM criteria might also be associated with the detection of macrovascular and microvascular complications in these patients. Therefore, this study’s main aim was to validate the use of GLIM criteria as a tool for diagnosing malnutrition in patients with T2DM compared to the SGA. In addition, we sought to investigate the association between GLIM criteria and vascular complications in these patients. 

## 2. Materials and Methods

### 2.1. Sample Selection and Study Design

This cross-sectional study was conducted in both the diabetes center and the nutrition outpatient clinic of the King Khalid University Hospital in Riyadh between February and August 2022. The study population comprised patients with T2DM diagnosed based on the guidelines of the American Diabetes Association, including both male and female patients, all of whom were aged between 18 and 65 years old. Excluded from the study were patients with type 1 diabetes, cancer, or psychiatric disorders; pregnant or lactating patients; and all patients <18 and >65 years of age. The King Saud University Ethics committee of Riyadh, KSA, approved the study (no. E-21-6446). Informed consent was obtained from all participants. MedCalc Statistical Software was used to determine an appropriate sample size based on an alpha level of 0.05 and a beta level of 0.10 (90% power); this resulted in a minimum expected area under the curve (AUC) of 0.70, a null hypothesis value of 0.5, and a ratio of well-nourished to malnourished of 1.87, based on a previous study conducted by Murillo and colleagues using the GLIM to determine nutritional status in diabetic patients [13]. The minimum sample size required was 92. We expected a response rate of 90%, which would result in a total sample size of 102.

### 2.2. Nutrition Screening and Assessment 

#### 2.2.1. Subjective Global Assessment 

The SGA was used to assess overall nutritional status, as described by Detsky et al. [10]. The SGA questionnaire included two types of questions. The first group of questions addressed patient history (weight changes in the past 6 months, dietary intake changes, gastrointestinal symptoms, functional capacity, as well as any diseases and their relation to nutritional requirements). The second group of questions was related to a physical examination (loss of subcutaneous fat, muscle wasting, and ankle edema). According to the SGA, patients can then be classified into three categories: category A (well-nourished), category B (moderately malnourished), or category C (severely malnourished). Due to the very small percentage of older adults classified as malnourished (about 3%), Categories B and C were combined into one group, denoted as the malnourished group.

#### 2.2.2. Nutrition Risk Screening 2002 

Patients were screened for malnutrition risk using the NRS-2002 [6], which was the first step for the GLIM criteria used in this study. The NRS-2002 consists of two steps. The first step is an initial screening that consists of yes–no questions about low BMI, weight loss, reduced dietary intake, and severe illness. If at least one of these questions is answered “yes”, the second step, which includes nutrition parameters and grading disease severity, is performed. Patients are then classified with a scoring system, with scores ≥3 indicating nutritional risk.

#### 2.2.3. GLIM Criteria

**Figure 1:**
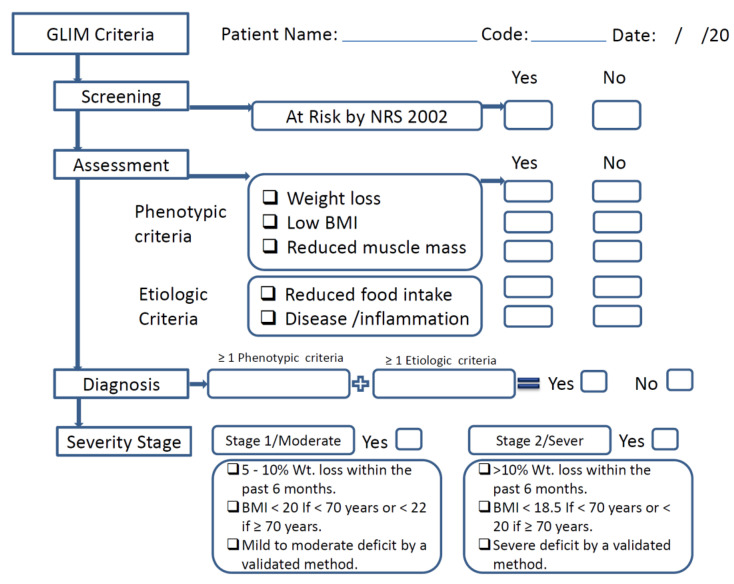
Schematic flow for the use of the GLIM in this study.

The GLIM contains both phenotypic criteria (nonvolitional weight loss, low BMI, and reduced muscle mass) and etiological criteria (reduced food intake or assimilation, acute disease/inflammation, and chronic disease) [12]. Fulfillment of at least one phenotypic criterion and etiologic criterion is necessary to diagnose malnutrition. The severity of the malnutrition is then determined based on threshold levels of the phenotypic criteria. These phenotypic criteria were considered to have been met when weight loss was >5% within the past 6 months or >10% beyond 6 months; when the patient’s BMI was low, according to the GLIM criteria for Asians (<18.5 kg/m^2^); or when reduced muscle mass was identified, according to appendicular skeletal muscle mass index (ASMMI), for which the cut-off value for men is <7 kg/m^2^ and for women is <5.7 kg/m^2^ [14]. For the etiologic criteria, the criterion “presence of inflammation or Disease severity” was ascertained according to serum albumin levels (<35 g/L) or neutrophil-to-lymphocyte ratio (NLR) (≥3 or <0.7) [15]. If laboratory data is unavailable, the presence of acute disease/injury or chronic disease can be used to guide the inflammatory assessment, as proposed by the GLIM group (Figure 1). 

The assessment of “reduced food intake or assimilation” relied on self-reported information about actual intake compared to usual intake (100%, 75%, 50%, 25%, or 0%), as did the presence of gastrointestinal symptoms (i.e., vomiting and diarrhea), or the presence of clinical diagnoses (i.e., pancreatitis, inflammatory bowel disease) that adversely impact food assimilation. Patients were then classified based on these phenotypic criteria into either Stage 1 (moderate malnutrition) or Stage 2 (severe malnutrition). In this study, moderate and severe malnutrition were grouped together into the single category ‘malnutrition’ for analysis [12].

### 2.3. Body Mass Index, Fat Mass, and Muscle Mass

Body weight and height were measured using a SECA weight scale (Seca co, Hamburg, Germany). BMI was calculated by dividing the patient’s weight in kilograms by the square of their height in meters (kg/m^2^). If the patient exhibited nonvolitional weight loss, the percentage of weight loss (WL %) was calculated by dividing their current weight by their previous weight and then multiplying the result by 100.

Bioelectric impedance analysis (BIA) was used to measure fat mass and muscle mass, measured using a TANITA MC-980MA (Tanita Corp., Tokyo, Japan). The participants stood barefoot on the scale after wiping their hands and feet with the water wipes to remove any substances that could affect the electrical current. Participants were also asked to remove any transmitters, such as mobile phones or smartwatches. The patient’s muscle mass was assessed based on their ASMMI, expressed as appendicular skeletal muscle mass (ASMM/height (m^2^)). ASMM was defined as the sum of the recorded muscle mass of the patient’s four limbs and was then used to calculate their ASMMI [16]. 

### 2.4. Biochemical Data and Information Related to T2DM 

Laboratory measurements of albumin and NLR were collected from the patients’ recent electronic medical records within 3 months of the assessment date. 

Information related to the duration of diabetes and diabetic complications (such as microvascular and macrovascular complications, and diabetic foot) was obtained directly from patients or their medical records.

### 2.5. Statistical Analysis

Statistical analysis was calculated using SPSS software version 22. The Shapiro–Wilk test was used to test the normality of the parameters. The continuous variables were expressed as mean ± standard deviation (SD) for normally distributed data or as a median and interquartile range (IQR) for data that are not normally distributed. The categorical variables were expressed as numbers (*n*) and percentages (%). Either Chi-square or Fisher tests were used for the categorical variables. For continuous data, the Student’s *t*-test was used for normally distributed continuous variables, and the Mann–Whitney U test was used for non-normally distributed continuous variables to compare patients with and without malnutrition.

Agreement between the tools was analyzed using the kappa coefficient (κ). The required value to establish the validity of GLIM was >0.80 [17]. The area under the receiver operating characteristic curve (AUC-ROC) with a confidence interval (CI) of 95%, as well as the measured sensitivity (Se), specificity (Sp), and both the predictive positive and predictive negative values (PPV and PNV, respectively) were determined to investigate the concurrent validity of the GLIM criteria, with the SGA serving as the reference method. 

Binary logistic regression analysis was used to determine possible associations between each criterion of the GLIM and micro- and macrovascular complications of Type 2 diabetes. Two models were applied for binary logistic regression; an unadjusted model (Model 1), and a second model (Model 2) which had been adjusted by age, gender, and duration of disease, in addition to other GLIM criteria. Each GLIM criterion was considered as an independent variable, and complications of T2DM were considered as the dependent variable (this is known as the enter method).

## 3. Results

### 3.1. Characteristics and Nutritional Status of Patients with T2DM

The general characteristics and nutritional status of the patients enrolled in this study are shown in Table S1. Out of a total of 101 T2DM patients, 57.4% were males with ages ranging from 26 to 65 years, 46% of patients had a university education, and most were married. Hypertension and dyslipidemia were ascertained to be the main comorbidities, present in 62.4% and 77.2% of patients, respectively. About half of the patients had microvascular complications, and 11.9% were active smokers.

Regarding nutritional status, use of the NRS-2002 found that 29.7% of the patients were at risk of malnutrition. According to GLIM criteria, 15.8% of the patients were diagnosed with malnutrition, which was quite similar to the results with the SGA (17.8%). 

### 3.2. Anthropometric Characteristics of Patients with T2DM According to the GLIM and SGA

Table 1 and Table 2 present the anthropometric status of the T2DM patients based on nutritional status using either the GLIM or SGA criteria, respectively. The results demonstrated a significant difference between the well-nourished group and the malnourished group in terms of BMI, muscle mass, fat mass, ASMM, and ASMMI when defined in accordance with GLIM criteria (Table 1), while there was a significant difference in ASMM between the two groups when using the SGA criteria (Table 2).

### 3.3. Comorbidities, Complications, and Medication of Patients with T2DM According to the GLIM Criteria and the SGA

According to the GLIM criteria, there were no significant differences in the comorbidities, complications, and medications used between the malnourished and well-nourished groups (Table 3). However, when nutritional status was assessed with the SGA, the percentage of patients diagnosed with hypothyroidism was significantly higher in the malnourished group (27.8%) compared to the well-nourished group (8.4%) (Table 4). In addition, according to the SGA, there were significantly more patients in the well-nourished group who used both oral antidiabetic medication and insulin (54.2%) than there were in the malnourished group (27.8%).

### 3.4. Concurrent Validity of GLIM Criteria

Table 5 presents the kappa (k) statistics and the AUC parameters of the GLIM. The GLIM showed substantial agreement with the SGA (κ = 0.778, *p* = 0.001). ROC analysis showed that the GLIM criteria had good sensitivity (77.8%) and excellent specificity (97.6%), with a comparable overall ability to detect malnutrition (Youden’s index = 0.754) to that of the SGA criteria. The AUC clearly showed the very good discriminative ability of the GLIM criteria to diagnose malnutrition (AUC = 0.877; 95% CI, 0.760–0.993). In addition, the PPV (the proportion of diabetic patients diagnosed as malnourished who were later confirmed to be malnourished) and the PNV (the proportion of those patients diagnosed as well-nourished who were likewise confirmed to be well-nourished) using the GLIM were 87.5% and 95.3%, respectively. 

### 3.5. Prevalence of GLIM Criteria in Patients with T2DM

Among the phenotypic criteria, nonvolitional weight loss was observed in about 17% of the patients, and reduced muscle mass was identified in 5.9%. Among the etiological criteria, reduced food intake or assimilation, as well as disease/inflammation, were observed in 36.6% and 44.6% of the patients, respectively. Regarding the phenotypic criteria, the percentage of patients with weight loss and reduced muscle mass was significantly higher in the malnourished group compared to the well-nourished group (87.5% vs. 3.5%, and 18.85% vs. 3.5, respectively) (Table 6). As for etiological criteria, both the percentage of patients with low food intake or assimilation, and the percentage of those with disease/inflammation, were significantly higher in the malnourished group compared to the well-nourished group (93.8% vs. 25.9, and 68.8% vs. 40%, respectively).

### 3.6. GLIM Criteria and T2DM Complications

Binary logistic regression analysis of each criterion of the GLIM in relation to macrovascular complications (Table 7) showed that the criterion most associated with macrovascular complications was disease burden/inflammation. This was true of both the unadjusted model (Model 1), (OR = 4.606; CI 1.5–14.0) and the adjusted model (Model 2) (OR = 4.266; CI 1.2–14.1). Other criteria had no significant effect with respect to macrovascular complications. In addition, Table 8 shows that there was no significant association between GLIM criteria and microvascular complications in Models 1 and 2.

## 4. Discussion

Malnutrition is one of the main complications associated with T2DM, and early detection of malnutrition, followed by its subsequent appropriate treatment, is highly important for patients’ health and glycemic control. Patients with T2DM usually have obesity as a common comorbidity. Malnutrition in a patient with T2DM and obesity goes frequently unrecognized as their fat mass masks an underlying loss of muscle mass [12]. According to ASPEN, type 2 diabetic patients with two or more manifestations such as weight loss, insufficient energy intake, subcutaneous fat tissue loss, muscle loss, regional or generalized edema, and decreased hand grip strength are malnourished [18]. Based on ESPEN, malnutrition include malnutrition without disease and disease-related malnutrition with or without inflammation in addition to overnutrition as another issue [19]. Patients with T2DM encounter a variable range of these malnutrition indicators. GLIM represents a core global leadership committee and a supporting interest group with representatives encountering global diversity and expertise in the diagnosis of malnutrition. The GLIM consensus recently provided a new two-step approach to the diagnosis of malnutrition. and called for validation studies in different clinical settings [20]. 

The SGA is recognized as the “semi-gold” standard by the GLIM criteria group [12]. Several studies have validated GLIM criteria in comparison with the SGA in different settings and populations [21,22,23]; however, until now, to the best of our knowledge, the GLIM has not been validated in patients with T2DM. For this reason, in the present study, GLIM was validated using the SGA as the reference standard in patients with T2DM, specifically.

One of the current study’s significant findings was the substantial agreement between the GLIM criteria and the SGA in diagnosing malnutrition in patients with T2DM. The GLIM criteria provided good accuracy in diagnosing malnutrition, with an AUC = 0.877, a sensitivity of 77.8%, and a specificity of 97.6%. 

The prevalence of malnutrition according to the GLIM criteria was found to be 15.8%, which was quite similar to the prevalence of malnutrition when measured according to the SGA (17.8%).

The agreement between the GLIM and SGA was κ = 0.778, which can be considered a good level of agreement. These findings were consistent with previous studies from different populations that validate GLIM criteria against the SGA. For example, Balci and his colleagues [21] compared GLIM with the SGA in a retrospective study of 271 adult patients who had been hospitalized with acute illnesses. The agreement they measured was κ = 0.804, with high sensitivity and specificity (86.05%, and 93.79%, respectively). In another prospective cohort study, the GLIM appears to have high accuracy in diagnosing malnutrition for adult/elderly hospitalized patients, with a high AUC = 0.842 and great sensitivity and specificity (86.6% and 81.6%, respectively); furthermore, the agreement between tools was substantial, with a κ = 64.5% [23]. By way of contrast, a retrospective cohort study involving 784 hospitalized patients found that the GLIM criteria presented a good specificity of 89.8% but a comparatively low sensitivity of 61.3%, using the SGA for comparison [24]. It is worth noting that due to a lack of information on reduced muscle mass as one of the criteria of the GLIM, the sensitivity was low; this was one of the limitations mentioned by the authors of the above study.

Recently, in a meta-analysis [25] of 20 studies to assess the accuracy of the GLIM criteria for diagnosing malnutrition (using various validated nutritional assessment tools, including the SGA, as reference standards), it was shown that GLIM criteria have high accuracy for diagnosing malnutrition, with a sensitivity of 0.72, a specificity of 0.82, and an AUC of 0.82. In addition, using the SGA as a gold standard, the study found that the GLIM had a better diagnostic value (sensitivity, 0.81; specificity, 0.80; AUC, 0.87) than other nutritional assessment tools.

Few studies have investigated the prevalence of malnutrition in patients with diabetes using GLIM criteria. A retrospective study performed on 159 elderly patients with T2DM found that 73% of the study population was at risk of malnutrition when using the Mini Nutritional Assessment Short Form (MNASF); in this population, 52.9% of patients were diagnosed with malnutrition according to the GLIM [26]. However, in our study, 15.8% were diagnosed with malnutrition. The comparatively high percentage of malnourished patients with T2DM found in the Sanz-París et al. study [26] could be due to the characteristics of the population enrolled in the study (being statistically older, on average), or to the nutritional assessment tool used (MNASF). Furthermore, in a prospective observational cohort study of 110 patients with diabetes, of which 84% had T2DM, malnutrition was identified in 24% of the patients according to the GLIM [27]. 

A major indication of malnutrition is weight loss, and the GLIM consensus strongly emphasizes this [12]. In the current study, the most prevalent phenotypic criterion in malnourished patients was weight loss (87.5%). This finding agreed with the Sanz-París et al. study [26], which also showed the most prevalent phenotypic criterion to be weight loss, measured at 54.7% in geriatric patients with T2DM. 

Another important phenotypic criterion in the GLIM is reduced muscle mass. Since muscle is the main organ for metabolizing glucose, decreased muscle mass increases insulin resistance [28]. In contrast, the higher activation of protein degradation pathways brought on by insulin resistance or T2DM is linked to an accelerated loss of muscle mass [29]. In this study, low muscle mass was found in 3.5% of the well-nourished group; however, this proportion increased to 18.8% in the malnourished group. This finding agreed with a study conducted on adult Korean T2DM patients, which found that 12% of patients had low muscle mass [30]. Therefore, an early assessment of muscle mass in patients with T2DM should be given priority. Another Korean study assessed skeletal muscle mass in patients with T2DM by using the BIA and found that low muscle mass was not associated with the incidence of chronic kidney disease (CKD). However, patients with sarcopenic obesity were associated with a high risk of CKD [31]. Suyoto et al. [32] found that skeletal muscle mass, assessed by BIA, was inversely correlated with the plasma level of high sensitivity C-reactive protein (hs-CRP), indicating a pro-inflammatory status in patients with T2DM and low muscle mass. Reduction of muscle mass which is frequently reported in patients with T2DM may result from functional disturbances of mitochondrial respiration of cells in the skeletal muscles which may lead to enhanced stimulation of skeletal muscle atrophy [33].

For the assessment of skeletal muscle mass, this study used BIA combined with anthropometric measurements to calculate the appendicular skeletal muscle mass index. However, there is a debate regarding the most accurate techniques for measuring reduced muscle mass, particularly in clinical settings. Based on this diversity and difficulty, the assessment of muscle mass in the clinical setting is less frequently measured than other phenotypic malnutrition criteria of the GLIM. Moreover, its interpretation may be more difficult and complicated especially in low resources settings and in settings that lack practitioners skilled in body composition analysis methods [14]. Recently, the commonly used techniques to estimate muscle mass includes dual-energy X-ray absorptiometry (DXA), computed tomography (CT), magnetic resonance imaging (MRI), and bioelectric impedance (BIA), as well as anthropometric measurements [34]. Besides, Ultrasound is a promising imaging technique that can measure muscle thickness and cross-sectional area in an easy and noninvasive manner. The main parameters of muscle architecture, such as muscle volume, fascicle length, and pennation angle can be also measured [35]. In 2022, a review by Sbrignadello et al. [36] suggested that BIA is a suitable metric for body composition analysis in instances of T2DM complicated by muscle loss. Nevertheless, to increase the accuracy of malnutrition diagnoses, standardizing appropriate methodologies for skeletal muscle mass measurement in clinical practice is highly recommended. It is highly recommended also to use validated population- and sex-specific cutoff values for each muscle measurement. Measurement of skeletal muscle function such as hand grip was not recommended as a surrogate measurement of muscle mass [14].

Regarding etiological criteria, reduced food intake was the most common criterion in malnourished patients, appearing in 93.8% of malnourished patients, while disease/inflammation appeared in 68.8% of patients. Inflammation was determined by measuring the level of NLR in the absence of albumin. NLR is widely used as a reliable and easily available inflammatory marker. The NLR has been suggested for possible use as a predictor of coronary artery disease, microvascular complications, and end-organ damage in patients with T2DM in an Indian study [37]. However, it is worth noting that low albumin is the parameter recommended by the GLIM group to indicate inflammation [12]. 

Our study showed that the prevalence of macrovascular complications was slightly higher in the malnourished group than in the well-nourished group when measured according to GLIM, but this was not found to be statistically significant. By comparison, the logistic regression results showed that the disease burden/inflammation criterion was significantly associated with macrovascular complications in patients with T2DM. It was reported that inflammatory markers such as IL-6 levels significantly predict macrovascular complications and mortality in patients with T2DM who have baseline cardiovascular disease or risk factors [38]. Moreover, high levels of CRP and IL-6 might be linked with increased coagulability and liability for thrombus formation in individuals with T2DM who have microvascular complications [39].

This was the first study that applied the GLIM criteria in T2DM patients using SGA as a comparator. However, there were some limitations to this study, in that it was a cross-sectional study collected in a single center in Riyadh. Moreover, due to a lack of laboratory information for some patients, food intake was assessed by self-report, rather than direct dietary assessment tools such as diet recall, diet diaries, or food frequency questionnaires.

## 5. Conclusions

To conclude, the GLIM criteria presented an acceptable level of validity, with similarly good sensitivity and specificity levels, and had a substantial agreement with the SGA. Furthermore, this study found an association between the GLIM inflammation criterion and macrovascular complications. 

Further validation studies on a nationwide scale using the SGA as a comparator are highly recommended in order to confirm these results in patients with T2DM. Furthermore, we highly recommend conducting GLIM-validation studies both in the KSA and abroad with different types of patients to investigate the possibility of using GLIM criteria as a standardized nutritional assessment.

## Figures and Tables

**Table 1:** Anthropometric characteristics of patients with T2DM according to the GLIM.

	GLIM			
Variable	All *n* = 101	Well-Nourished (*n* = 85)	Malnourished (*n* = 16)	*p* Value
Anthropometric				
BMI	28.4 ± 4.8	29.07 ± 4.61	24.86 ± 4.7	0.001
Muscle mass	49.7 ± 8.56	50.54 ± 8.4	45.27 ± 8.2	0.023
Fat mass	23.89 ± 10.6	25.16 ± 10.5	17.13 ± 8.6	0.005
ASMM	22.07 ± 4.36	22.5 ± 4.26	19.73 ± 4.28	0.019
ASMMI	8.19 (2)	8.40 (1)	7.45 (1)	0.008

BMI: body mass index; ASMM: appendicular skeletal muscle mass; ASMMI: appendicular skeletal muscle mass index. Data presented as mean ± SD or median and IQR. Significance at *p*-value < 0.05.

**Table 2:** Anthropometric characteristics of patients with T2DM according to the SGA.

	SGA			
Variable	All *n* = 101	Well-Nourished (*n* = 83)	Malnourished (*n* = 18)	*p* Value
Anthropometrics				
BMI	28.4 ± 4.8	28.83 ± 4.6	26.45 ± 5.3	0.060
Muscle mass	49.7 ± 8.56	50.41 ± 8.35	46.45 ± 9.0	0.075
Fat mass	23.89 ± 10.6	24.77 ± 10.7	19.83 ± 9.32	0.075
ASMM	22.07 ± 4.36	22.47 ± 4.22	20.24 ± 4.64	0.049
ASMMI	8.19 (2)	8.40 (2)	7.90 (2)	0.112

BMI: body mass index; ASMM: appendicular skeletal muscle mass; ASMMI: appendicular skeletal muscle mass index. Data presented as mean ± SD or median and IQR. Significance at *p*-value < 0.05.

**Table 3:** Comorbidities, complications, and medication in patients with T2DM according to the GLIM.

	GLIM			
Variable	All *n* = 101	Well-Nourished (*n* = 85)	Malnourished (*n* = 16)	*p* Value
Comorbidities				
HTN	63 (62.4%)	(60%) 51	12 (75%)	0.399
DLP	78 (77.2%)	(76.5%) 65	(81.3%) 13	1.00
Hypothyroidism	12 (11.9%)	8 (9.4%)	4 (25%)	0.095
IDA	7 (6.9%)	6 (7.1%)	1 (6.3%)	1.00
Asthma	5 (5%)	4 (4.7%)	1 (6.3%)	0.586
Other	39 (38.6%)	33 (38.8%)	6 (37.5%)	1.00
Macrovascular complications	19 (18.8%)	15 (17.6%)	4 (25%)	0.495
Microvascular complications	50 (49.5%)	41 (48.2%)	9 (56.3%)	0.596
Neuropathy	27 (26.7%)	24 (28.2%)	3 (18.8%)	0.548
Nephropathy	15 (14.9%)	11 (12.9%)	4 (25%)	0.250
Retinopathy	27 (26.7%)	22 (25.9%)	5 (31.3%)	0.759
Medication				
Oral antidiabetic	98 (97.0%)	82 (96.5%)	16 (100%)	1.00
Insulin	54 (53.5%)	48 (56.5%)	6 (37.5%)	0.163
Both	50 (49.5%)	44 (51.8%)	6 (37.5%)	0.295

HTN: hypertension; DLP: dyslipidemia; IDA: iron deficiency anemia. Data presented as *n* (%). Significance at *p*-value < 0.05.

**Table 4:** Comorbidities, complications, and medication in patients with T2DM according to the SGA.

	SGA			
Variable	All *n* = 101	Well-Nourished (*n* = 83)	Malnourished (*n* = 18)	*p* Value
Comorbidities				
HTN	63 (62.4%)	49 (59.0%)	14 (77.8%)	0.183
DLP	78 (77.2%)	63 (75.9%)	15 (83.3%)	0.757
Hypothyroidism	12 (11.9%)	7 (8.4%)	5 (27.8%)	0.021
IDA	7 (6.9%)	6 (7.2%)	1 (5.6%)	1.00
Asthma	5 (5%)	4 (4.8%)	1 (5.6%)	1.00
Other	39 (38.6%)	32 (38.6%)	7 (38.9%)	0.979
Macrovascular complications	19 (18.8%)	16 (19.3%)	3 (16.7%)	1.00
Microvascular complications	50 (49.5%)	39 (47.0%)	11 (61.1%)	0.277
Neuropathy	27 (26.7%)	23 (27.7%)	4 (22.2%)	0.774
Nephropathy	15 (14.9%)	11 (13.3%)	4 (22.2%)	0.462
Retinopathy	27 (26.7%)	20 (24.1%)	7 (38.9%)	0.199
Medication				
Oral antidiabetic	98 (97.0%)	80 (96.4%)	18 (100%)	1.00
Insulin	54 (53.5%)	48 (57.8%)	6 (33.3%)	0.059
Both	50 (49.5%)	45 (54.2%)	5 (27.8%)	0.042

HTN: hypertension; DLP: dyslipidemia; IDA: iron deficiency anemia. Data presented as *n* (%). Significance at *p*-value < 0.05.

**Table 5:** Concurrent validity of GLIM criteria, using the SGA as reference.

Statistical Parameters of Concurrent Validity			
Kappa (κ)	0.788		(*p* = 0.001)
AUC (95% CI)	0.877	(0.760–0.993)	(*p* = 0.001)
Sensitivity	77.8%		
Specificity	97.6%		
Predictive positive value	87.5%		
Predictive negative value	95.3%		
Youden’s index	0.754		

AUC: area under the curve; CI: confidence interval. Significance at *p*-value < 0.05.

**Table 6:** Prevalence of phenotypic and etiologic GLIM criteria among patients with T2DM.

	All *n* = 101	Well-Nourished (*n* = 85)	Malnourished (*n* = 16)	*p* Value
Phenotypic criteria				
Weight loss	17 (16.8%)	3 (3.5%)	14 (87.5%)	0.001
Low BMI	1 (1%)	0	(6.3%) 1	0.158
Reduce muscle mass	6 (5.9%)	3 (3.5%)	3 (18.8%)	0.049
Etiologic Criteria				
Low food intake	37 (36.6%)	22 (25.9%)	15 (93.8%)	0.001
Disease/inflammation	45 (44.6%)	34 (40%)	11 (68.8%)	0.034

BMI = Body mass index. Data for categorical variables are expressed as numbers and percentages (%). Significance at *p*-value < 0.05.

**Table 7:** Binary logistic regression for the association between GLIM criteria and the prevalence of macrovascular complications.

	Model 1		Model 2	
Variable	OR (95% CI)	*p* Value	OR (95% CI)	*p* Value
Weight loss	2.08 (0.633–6.85)	0.227	1.563 (0.38–6.44)	0.536
Low BMI	0.92 (0.824–1.03)	0.160	0.941 (0.83–1.07)	0.349
Reduce muscle mass	0.86 (0.094–7.78)	0.890	0.744 (0.06–9.97)	0.824
Reduce food intake	1.74 (0.632–4.76)	0.284	0.898 (0.25–3.16)	0.867
Disease burden/inflammation	4.606 (1.511–14.04)	0.007	4.266 (1.29–14.11)	0.017

OR: odds ratio; CI: confidence interval; Model 1: crude; Model 2: adjusted for age, gender, disease duration, and other GLIM criteria. Significance at *p*-value < 0.05.

**Table 8:** Binary logistic regression for the association between GLIM criteria and the prevalence of microvascular complications.

	Model 1		Model 2	
Variable	OR (95% CI)	*p* Value	OR (95% CI)	*p* Value
Weight loss	1.57 (0.546–4.52)	0.402	1.22 (0.38–3.96)	0.737
Low BMI	0.98 (0.905–1.06)	0.652	0.99 (0.90–1.08)	0.767
Reduce muscle mass	0.49 (0.086–2.80)	0.422	0.43 (0.07–2.79)	0.378
Reduce food intake	1.58 (0.701–3.58)	0.269	1.37 (0.53–3.51)	0.515
Disease burden/inflammation	1.82 (0.82–4.03)	0.138	1.58 (0.68–3.68)	0.289

OR: odds ratio; CI: confidence interval; Model 1: crude; Model 2: adjusted for age, gender, disease duration, and other GLIM criteria. Significance at *p*-value < 0.05.

## Data Availability

The raw data supporting the conclusions of this article will be made available by the authors, without undue reservation, to any qualified researcher.

## Supplementary Materials

The following supporting information can be downloaded at: https://www.mdpi.com/article/10.3390/nu15040897/s1, Table S1. Characteristics and nutritional status of study participants.
