# Supplementary material for: GLIM Criteria for Assessment of Malnutrition in Saudi Patients with Type 2 Diabetes
Source: Nutrients. 2023 Feb 10;15(4):897. doi: 10.3390/nu15040897 (PMC9959813; doi:10.3390/nu15040897)
Supplement: Supplementary file 1 [file nutrients-15-00897-s001.zip › nutrients-2157042-supplementary.pdf]

Table S1. Characteristics and nutritional status of study participants.

Variable	Total n = 101
Age	56.50 (13)
Gender	
Male	58 (57.4 %)
Female	43 (42.6 %)
Academic level	
Primary School	16 (15.8 %)
Middle school	13 (12.9 %)
High School	25 (24.8 %)
University	47 (46.5 %)
Material Status	
Single	5 (5 %)
Married	90 (89.1 %)
Divorced	3 (3 %)
Widowed	3 (3 %)
Duration of DM (years)	14.6 ± 7.81
Smoking	12 (11.9 %)
Comorbidities	
HTN	63 (62.4 %)
DLP	78 (77.2 %)
Hypothyroidism	12 (11.9 %)
IDA	7 (6.9 %)
Asthma	5 (5 %%
Other	39 (38.6 %)
Cardiovascular disease	19 (18.8 %)
Microvascular complication	50 (49.5 %)
Neuropathy	27 (26.7 %)
Nephropathy	15 (14.9 %)
Retinopathy	27 (26.7 %)
Medication	
Oral antidiabetic	98 (97.0 %)
Insulin	54 (53.5 %)
Both	50 (49.5 %)
Nutritional status	
GLIM	
Well-nourished	85 (84.2%)
Malnourished	16 (15.8%)
SGA	
Well-nourished	83 (82.2%)
Malnourished	18 (17.8%)
NRS-2002	

At risk of malnutrition	30 (29.7%)
Not at risk of malnutrition	71 (70.3 %)

HTN: Hypertension; DLP: Dyslipidemia; IDA: Iron Deficiency Anemia; GLIM: Global Leadership Initiative on Malnutrition; SGA: Subjective Global Assessment; NRS-2002: Nutrition Risk Screening 2002. Data presented as median & (IQR) or as number and percentage (%).
